# Rapid mono and biexponential 3D-T_1ρ_ mapping of knee cartilage using variational networks

**DOI:** 10.1038/s41598-020-76126-x

**Published:** 2020-11-05

**Authors:** Marcelo V. W. Zibetti, Patricia M. Johnson, Azadeh Sharafi, Kerstin Hammernik, Florian Knoll, Ravinder R. Regatte

**Affiliations:** 1 Bernard and Irene Schwartz Center for Biomedical Imaging, New York University School of Medicine, 660 1st Ave, 4th Floor, New York, NY 10016 USA; 2 Department of Computing, Imperial College London, London, UK

**Keywords:** Magnetic resonance imaging, Three-dimensional imaging

## Abstract

In this study we use undersampled MRI acquisition methods to obtain accelerated 3D mono and biexponential spin–lattice relaxation time in the rotating frame (T_1ρ_) mapping of knee cartilage, reducing the usual long scan time. We compare the accelerated T_1ρ_ maps obtained by deep learning-based variational network (VN) and compressed sensing (CS). Both methods were compared with spatial (S) and spatio-temporal (ST) filters. Complex-valued fitting was used for T_1ρ_ parameters estimation. We tested with seven in vivo and six synthetic datasets, with acceleration factors (AF) from 2 to 10. Median normalized absolute deviation (MNAD), analysis of variance (ANOVA), and coefficient of variation (CV) were used for analysis. The methods CS-ST, VN-S, and VN-ST performed well for accelerating monoexponential T_1ρ_ mapping, with MNAD around 5% for AF = 2, which increases almost linearly with the AF to an MNAD of 13% for AF = 8, with all methods. For biexponential mapping, the VN-ST was the best method starting with MNAD of 7.4% for AF = 2 and reaching MNAD of 13.1% for AF = 8. The VN was able to produce 3D-T_1ρ_ mapping of knee cartilage with lower error than CS. The best results were obtained by VN-ST, improving CS-ST method by nearly 7.5%.

## Introduction

Quantitative mapping using the spin–lattice relaxation time in the rotating frame (T_1ρ_) has shown to be useful for early detection of osteoarthritis (OA)^1^, since T_1ρ_ mapping is sensitive to the proteoglycan content of the cartilage^2^. Biexponential relaxation models^3^ can provide more specific information about the water compartments of the various structures in the extracellular matrix of the cartilage^4^. However, in order to produce T_1ρ_ maps, many T_1ρ_ weighted images must be acquired, taking a long acquisition time, especially if biexponential models are desired^3^. Predominantly, monoexponential models are used for OA, but a recent study^5^ suggested that biexponential mapping of cartilage can provide better diagnostic performance. Studies on cartilage degradation^6^ show that the loss of macromolecules changes the distribution of multiexponential relaxation components. In^3,7,8^ it was observed biexponential relaxation in the knee cartilage of healthy volunteers in T_1ρ_ and T_2_ mappings.

Reducing the acquisition time of T_1ρ_ mapping is essential for practical use. Recently, compressive sensing (CS) acceleration has been studied for monoexponential T_1ρ_^9–11^ and biexponential T_1ρ_ mapping^12^ of cartilage. These studies demonstrated that CS combined with 3 × 3 filtering can reduce acquisition time by 10 times, with an error of 6.5% for monoexponential models^11^ and 15% for biexponential models^12^ (errors are 10% and 20% for accelerated acquisitions of 6 times if no filtering is used). Clearly, previous studies showed that the biexponential T_1ρ_ mapping error using CS is much higher than that of monoexponential T_1ρ_ mapping error for the same acceleration factor, usually because biexponential mapping is a more ill-posed problem, and more sensitive to noise and residual artifacts.

One possible approach to improve image and parametric mapping quality is to use deep learning-based reconstruction methods^13^ such as Variational Networks (VNs)^14^. A VN is an unrolling-based deep MR image reconstruction approach developed for multi-coil imaging^15^, that combines a gradient-like iterative algorithm with learned regularization, providing a reconstruction specifically tailored for a particular set of images, in this case, for knee T_1ρ_-weighted images. In general, the trained regularizing filters, activation functions, and other network parameters provide the missing information, due to k-space undersampling, for the reconstruction process. Also, the VN uses relatively fast algorithmic implementation based on convolutional layers, enabling faster reconstructions than typical iterative algorithms used in CS. However, this comes at the cost of formulating the image reconstruction problem into a highly non-linear and non-convex optimization problem. This opens the question if the found local minimum generalizes to different types of data. Up to this point, all studies that investigated the properties of this approach^14,16–18^ have focused on qualitative imaging where the actual signal values of the reconstructed images are arbitrarily defined. It is, therefore, an open question whether such a reconstruction method can also be used for the estimation of quantitative biomarkers, where systematic deviations in signal intensities of the reconstructed images lead to erroneous parameter maps.

In this study, we compare the VN, trained with real and synthetically generated knee cartilage images, against CS approaches for mono and biexponential T_1ρ_ mapping^11,12^. It is not our intention here to compare different deep learning methods for image reconstruction^13,15,19,20^, but compare one good representative of this class against one good representative of CS, which is among the current state-of-the-art methods for T_1ρ_ mapping^11,12,21^. In order to have a fair comparison between these approaches, the VN and CS used the same pre-available data for training or tuning the parameters of the algorithms. Also, in both methods, the comparisons involved models using only spatial (S) information (2D, time-independent image reconstructions) and spatio-temporal (ST) information (2D + time, the whole sequence of T_1ρ_–weighted images jointly reconstructed). After reconstruction, complex-valued fitting is used to find the T_1ρ_ mapping parameters for mono and biexponential models.

## Results

**Figure 1:**
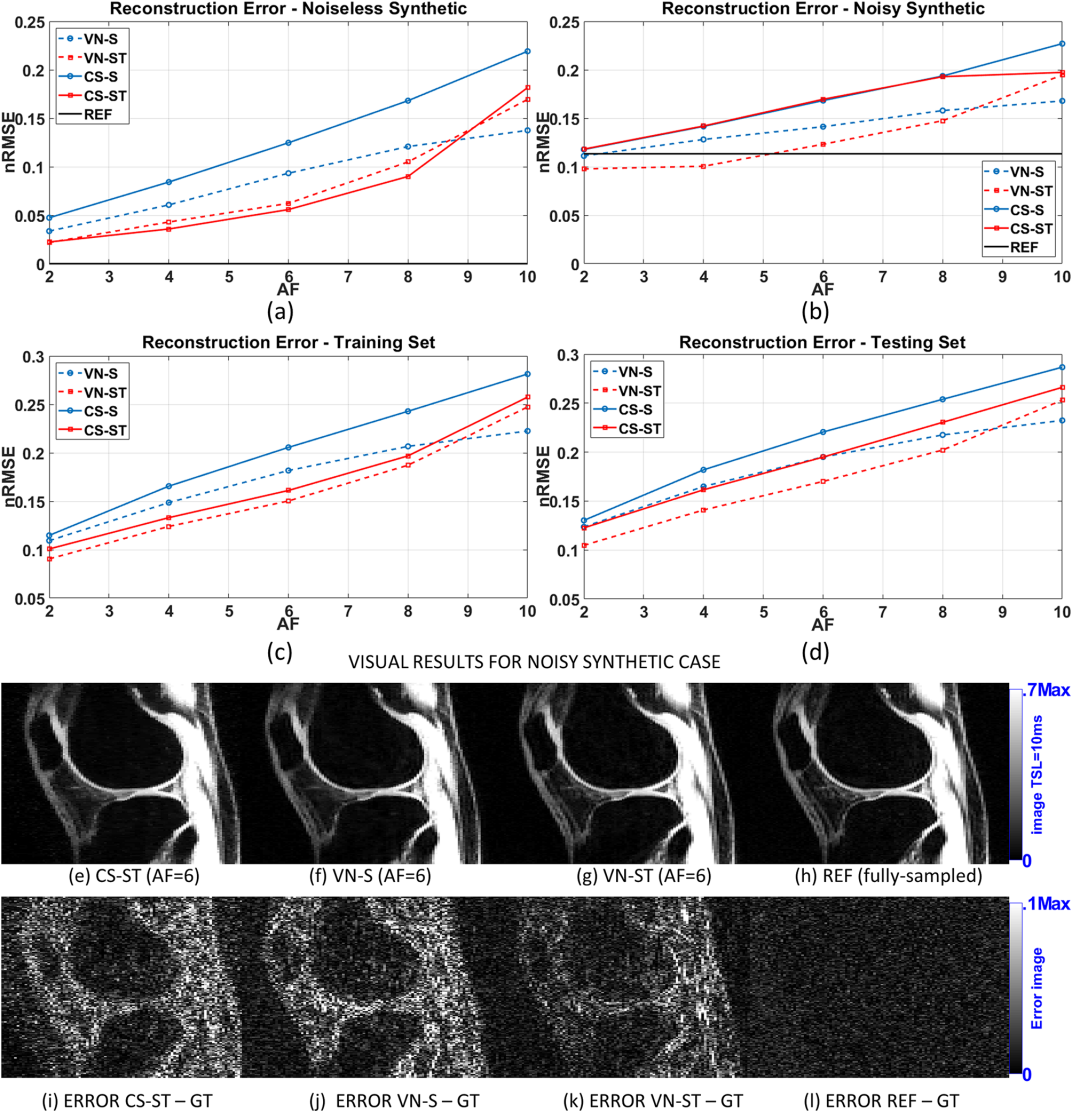
Comparison of the reconstruction error (nRMSE) using (**a**) only the noiseless synthetic dataset, (**b**) only the noisy synthetic dataset, (**c**) all training datasets, and (**d**) all testing datasets. Representative reconstructed images of the noisy synthetic case, using AF = 6, are shown in (**e**)–(**g**) and the fully-sampled reference in (**h**), and their voxel-wise absolute difference against the ground truth is shown in (**i**)–(**l**).

The comparison of the reconstruction errors, assessed using normalized root mean squared error (nRMSE), is shown in Fig. 1. The results for the noiseless and noisy synthetic data, where the ground truth (GT) is known, are shown in Fig. 1(a)–(b). In those plots, the reference method (fully-sampled reconstruction, denoted as REF) can also be compared with the GT, as well as the accelerated methods. The results for the knee cartilage training and testing group are shown in Fig. 1(c)–(d). In those plots, the accelerated methods are compared against the reference, since no GT is known for all knee cartilage images. Representative reconstructed images of the noisy synthetic case are shown in Fig. 1(e)–(h) their error against the ground truth is shown in Fig. 1(i)–(l).

In Fig. 1 we can observe that VN-S always outperforms CS-S, and VN-ST outperforms CS-ST most of the time. The only observed exception was the noiseless synthetic case. It is interesting to observe in Fig. 1(b) that VN-ST had a denoising effect and performed better than the noisy fully-sampled reference for AF ≤ 4. In Fig. 1(l) we can see that the error of the REF is basically noise.

**Figure 2:**
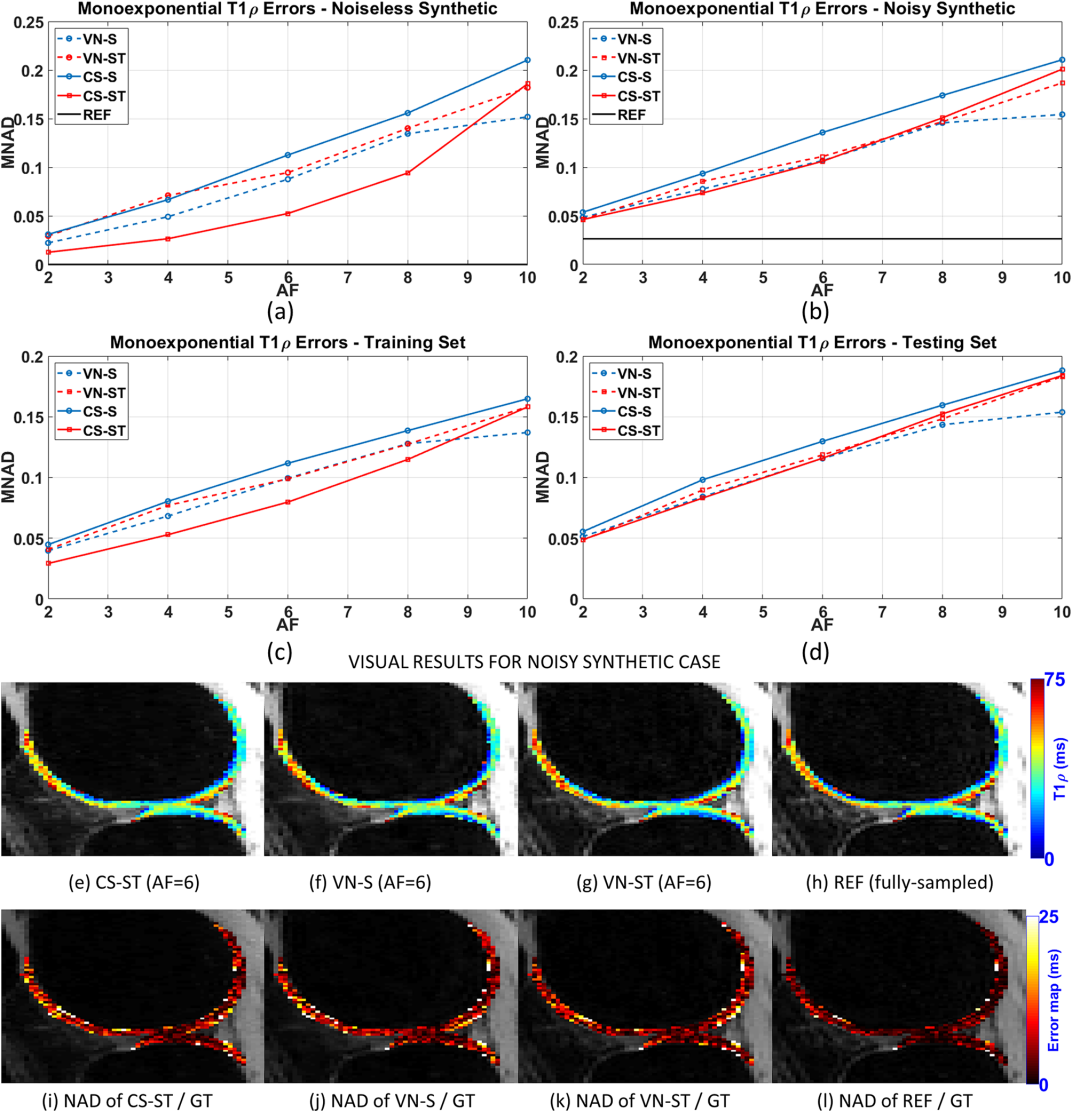
Comparison of the monoexponential T_1ρ_ mapping error (MNAD) using (**a**) only the noiseless synthetic dataset, (**b**) only the noisy synthetic dataset, (**c**) all the training datasets, and (**d**) all the testing datasets. Representative monoexponential T_1ρ_ maps of the noisy synthetic case, using AF = 6, are shown in (**e**)–(**g**), and the fully-sampled reference in (**h**), and their error (NAD) against the ground truth maps are shown in (**i**)–(**l**).

In Fig. 2, the comparison of the monoexponential T_1ρ_ mapping errors, given by the MNAD, is shown. The results for the noiseless and noisy synthetic data, where the GT is known, are shown in Fig. 2(a)–(b). In those plots, all the maps can be compared with GT. The results for the training and testing group are shown in Fig. 2(c)–(d). In those plots, the maps from the accelerated methods are compared against the map obtained from the reference, since no GT maps are available for all the knee cartilage data. Representative monoexponential T_1ρ_ maps of the noisy synthetic case are shown in Fig. 2(e)–(h) their error (NAD) against the ground truth is shown in Fig. 2(i)–(l).

In Fig. 2 we can observe that CS-ST performed well in the noiseless case and with training data. However, the most relevant practical case is with synthetic noisy data and with testing data. In these cases, VN-S, VN-ST, and CS-ST performed equally well for monoexponential fitting. In all cases, VN-S outperformed CS-S for all AF.

**Figure 3:**
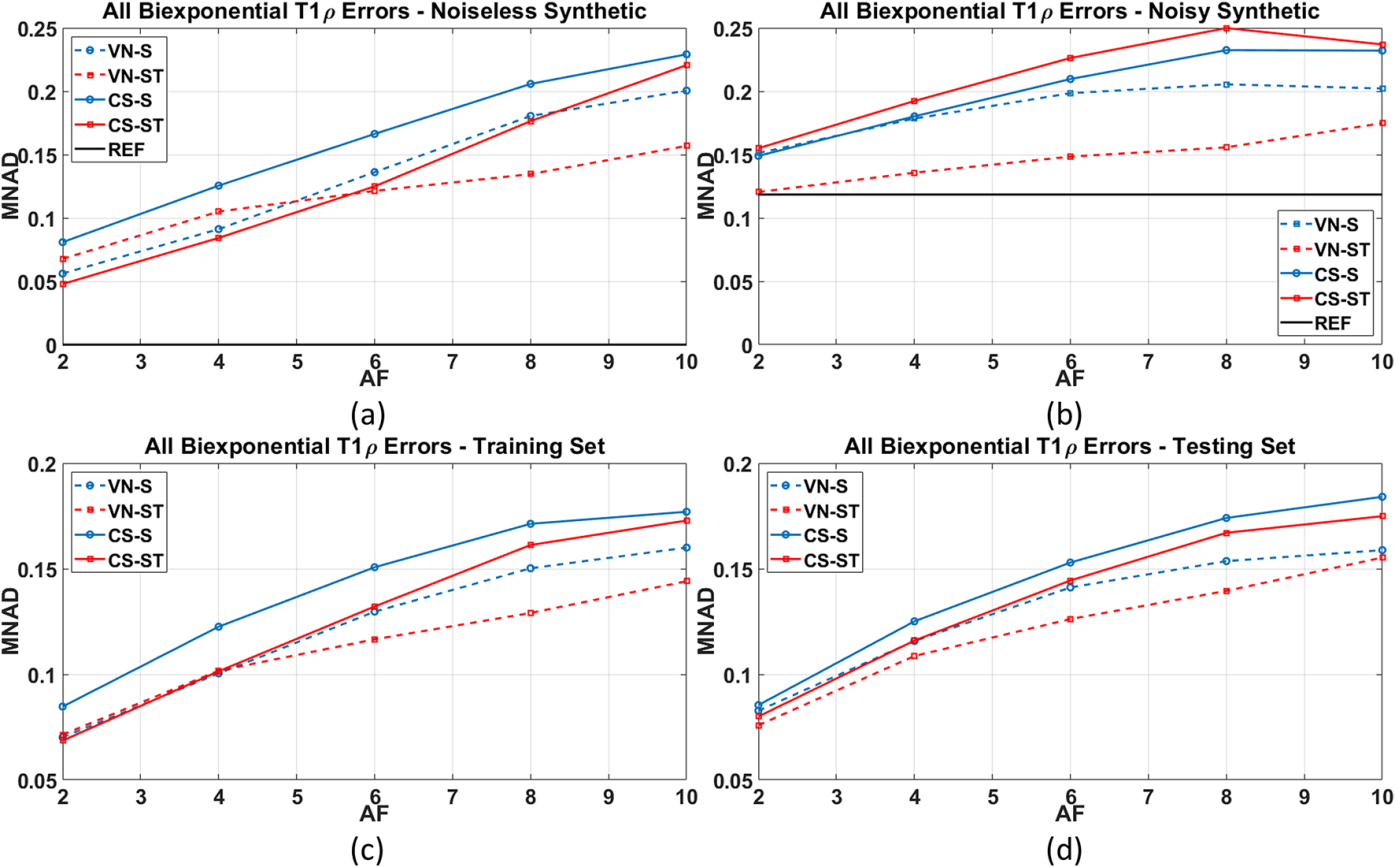
Comparison of the biexponential T_1ρ_ mapping error (MNAD) using (**a**) only the noiseless synthetic dataset, and (**b**) using only the noisy synthetic dataset, in (**c**) all the training datasets, and (**d**) all the testing datasets.

The results of comparing the biexponential T_1ρ_ mapping errors, given by the MNAD, are shown in Fig. 3. The results for the noiseless and noisy synthetic data, where the GT is known, are shown in Fig. 3(a)–(b). The results for the training and testing group are shown in Fig. 3(c)–(d).

In Fig. 3 we can observe that VN-ST performed better than the other methods most of AF in almost all cases, especially for high AF. This is extremely important to make biexponential T_1ρ_ mapping practical since it is more sensitive to noise and artifacts than monoexponential mapping^12,21,22^. In Table 1, we included the results with in vivo data. Table 1B shows the improved MNAD provided by VN-ST with biexponential mapping.

**Table 1:** MNAD of the T_1ρ_ maps for (A) monoexponential models using in vivo knee cartilage (testing and training) data and (B) biexponential models using in vivo knee cartilage data.

AF 2		AF4		AF 6		AF8		AF 10	
**(A) MNAD of monoexponential mapping of knee cartilage data**									
**CS-ST**	**0.050**	**CS-ST**	**0.080**	**CS-ST**	**0.106**	VN-S	0.130	VN-S	0.138
**VN-ST**	**0.051**	**VN-S**	**0.083**	**VN-ST**	**0.108**	VN-ST	0.130	CS-ST	0.156
**VN-S**	**0.054**	**VN-ST**	**0.085**	**VN-S**	**0.110**	CS-ST	0.134	CS-S	0.157
**CS-S**	**0.055**	**CS-S**	**0.091**	**CS-S**	**0.117**	CS-S	0.137	VN-ST	0.158
**(B) MNAD of biexponential mapping of knee cartilage data**									
**VN-ST**	**0.074**	**VN-ST**	**0.104**	**VN-ST**	**0.120**	VN-ST	0.131	VN-ST	0.143
**CS-ST**	**0.077**	**VN-S**	**0.107**	VN-S	0.128	VN-S	0.140	VN-S	0.147
**VN-S**	**0.078**	**CS-ST**	**0.108**	CS-ST	0.132	CS-ST	0.150	CS-ST	0.153
**CS-S**	**0.081**	**CS-S**	**0.118**	CS-S	0.141	CS-S	0.155	CS-S	0.157

Bold-marked results represent CS methods and corresponding AF that obtained MNAD below 12% on monoexponential and biexponential models.

The most relevant results for practical purposes are in Figs. 1(d), 2(d), and Fig. 3(d), where it is shown the performance with unseen testing data, which represents the expected performance with newly captured data.

**Figure 4:**
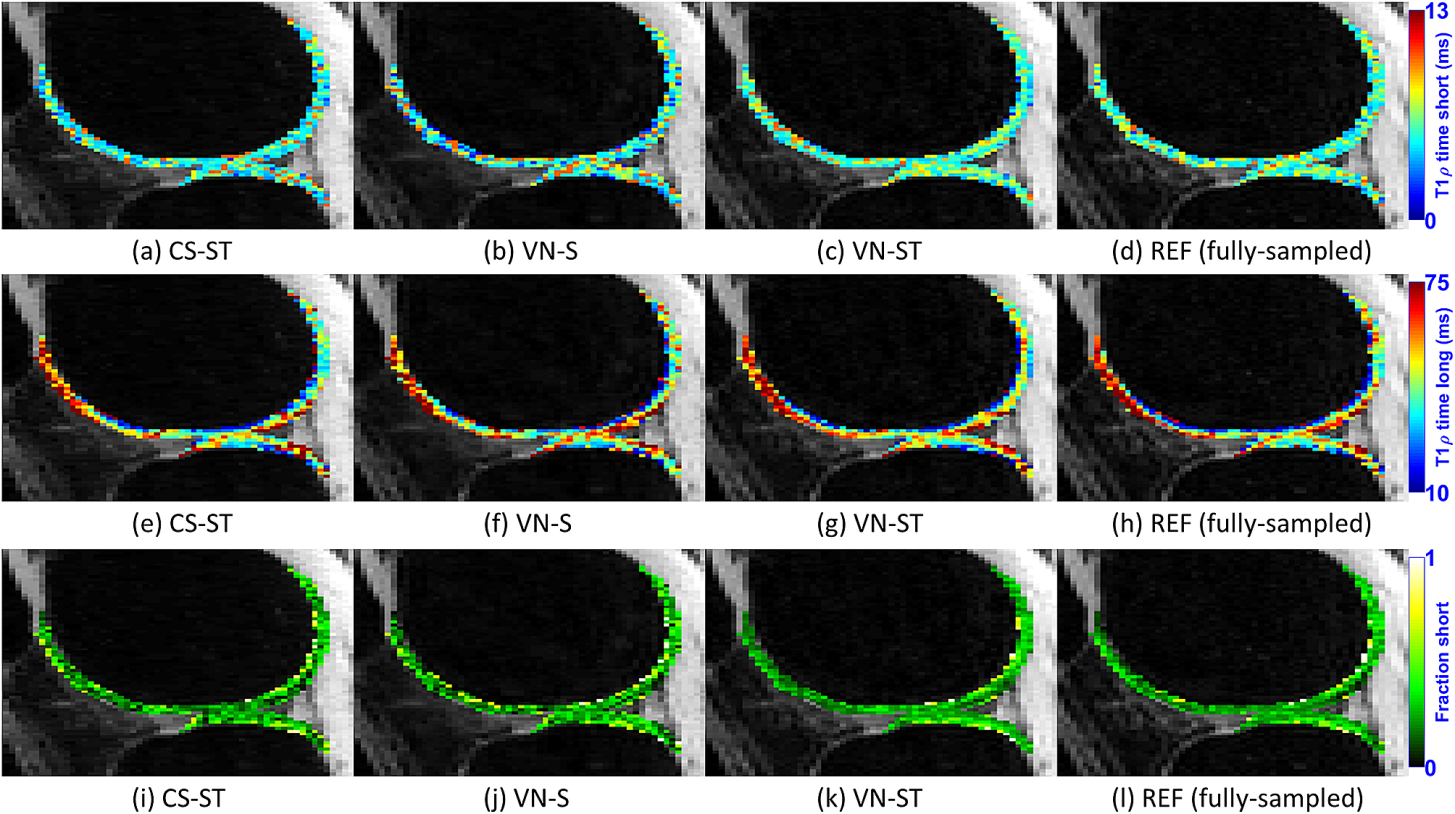
Representative biexponential T_1ρ_ maps, including short time (**a**)–(**d**), long time (**e**)–(**h**), and the fraction of the short component (**i**)–(**l**) for the noisy synthetic case, using AF = 6 on CS-ST, VN-S, and VN-ST, and using the fully-sampled reconstruction as reference.

Figure 4 shows representative maps for short time, in Fig. 4(a)–(d), for long times, in Fig. 4(e)–(h), and for short fractions, in Fig. 4(i)–(l), for the synthetic noise case. REF is fully sampled, and VN and CS methods use AF = 6.

**Figure 5:**
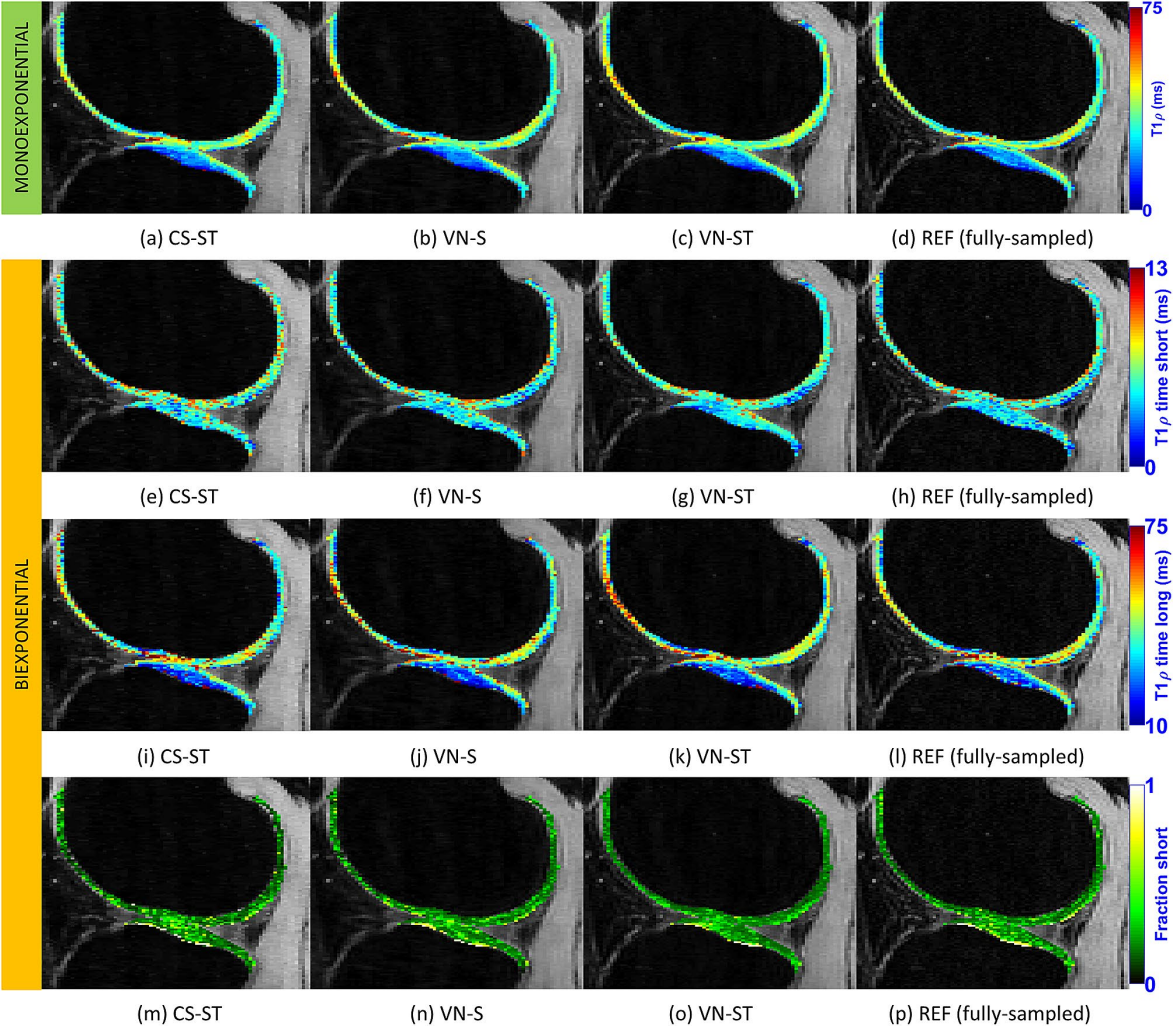
Representative monoexponential and biexponential T_1ρ_ maps, including monoexponential time (**a**)–(**d**), biexponential short time (**e**)–(**h**), long time (**i**)–(**l**), and the fraction of the short component (**m**)–(**p**) for the in vivo testing data. REF uses fully-sampled data, the VN and CS methods use AF = 6.

In Fig. 5, in vivo representative maps for monoexponential times are shown in Fig. 5(a)–(d), and for biexponential short time, in Fig. 5(e)–(h), for long time, in Fig. 5(i)–(l), and for short fractions, in Fig. 5(m)–(p). Also, REF is fully sampled, and VN and CS methods use AF = 6.

**Figure 6:**
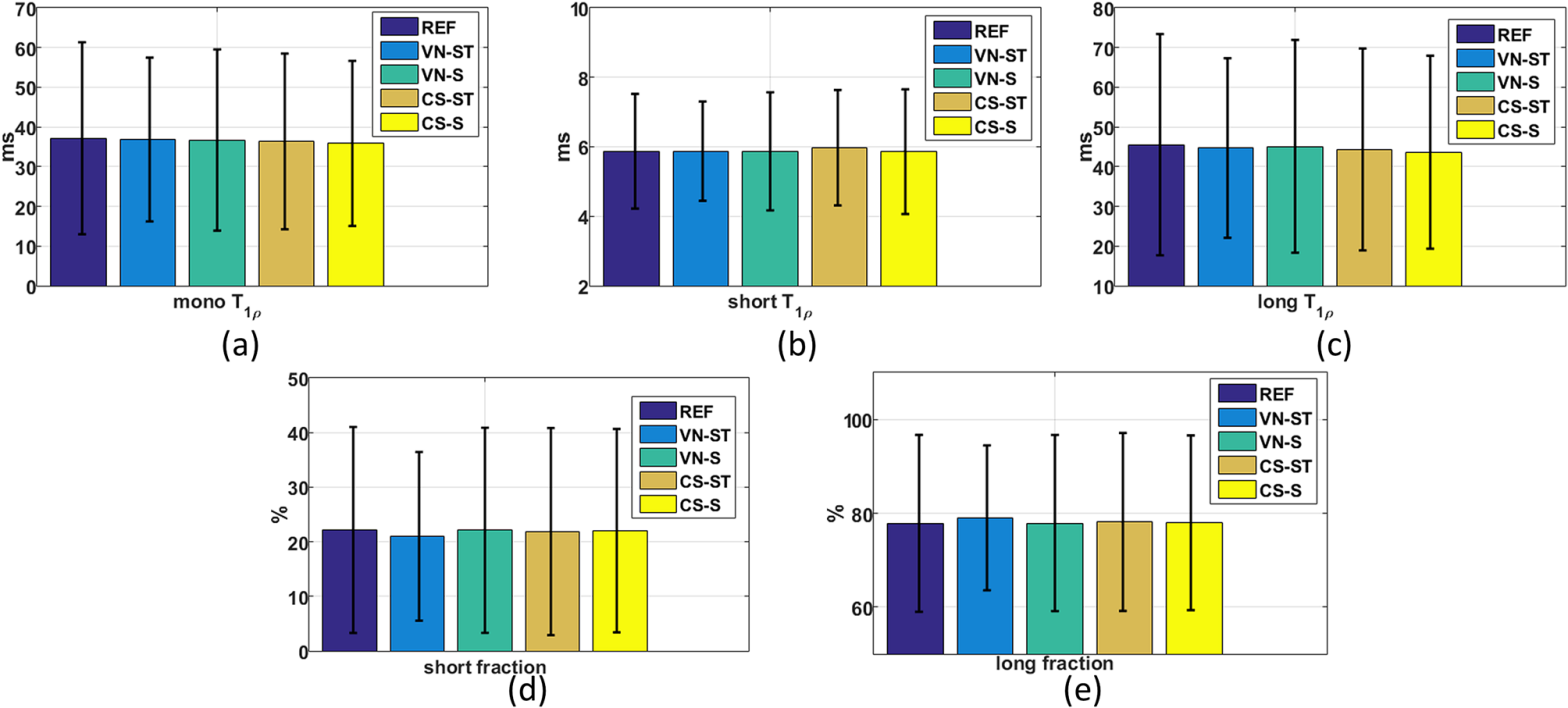
Central tendency (mean, Eq. (11)) represented by the coloured bars, variability (standard deviation, Eq. (12)) represented by whiskers, for (**a**) monoexponential T_1ρ_ values (in ms) for (**b**) biexponential short T_1ρ_ time (in ms), (**c**) biexponential long T_1ρ_ time (in ms), (**d**) biexponential short fraction (in %), and (**e**) biexponential long fraction (in %). The REF is fully sampled and the other methods (VN and CS) used AF = 4.

In Fig. 6, one can see that the central tendency (mean) and variability (SD) of the exponential parameters of the in vivo knee cartilage, for all ROIs, for AF = 4. This information is also included by an individual ROI basis in the Supplementary Tables S1–S5. The p-values of the ANOVA in Supplementary Tables S1–S5 indicate that any difference in the mean values of the parameters obtained by the various accelerated methods is due to chance. This result means that the accelerated methods do not introduce any bias in the central tendency of the model. In addition, there is smaller variability in all accelerated methods when compared to fully sampled REF.

**Figure 7:**
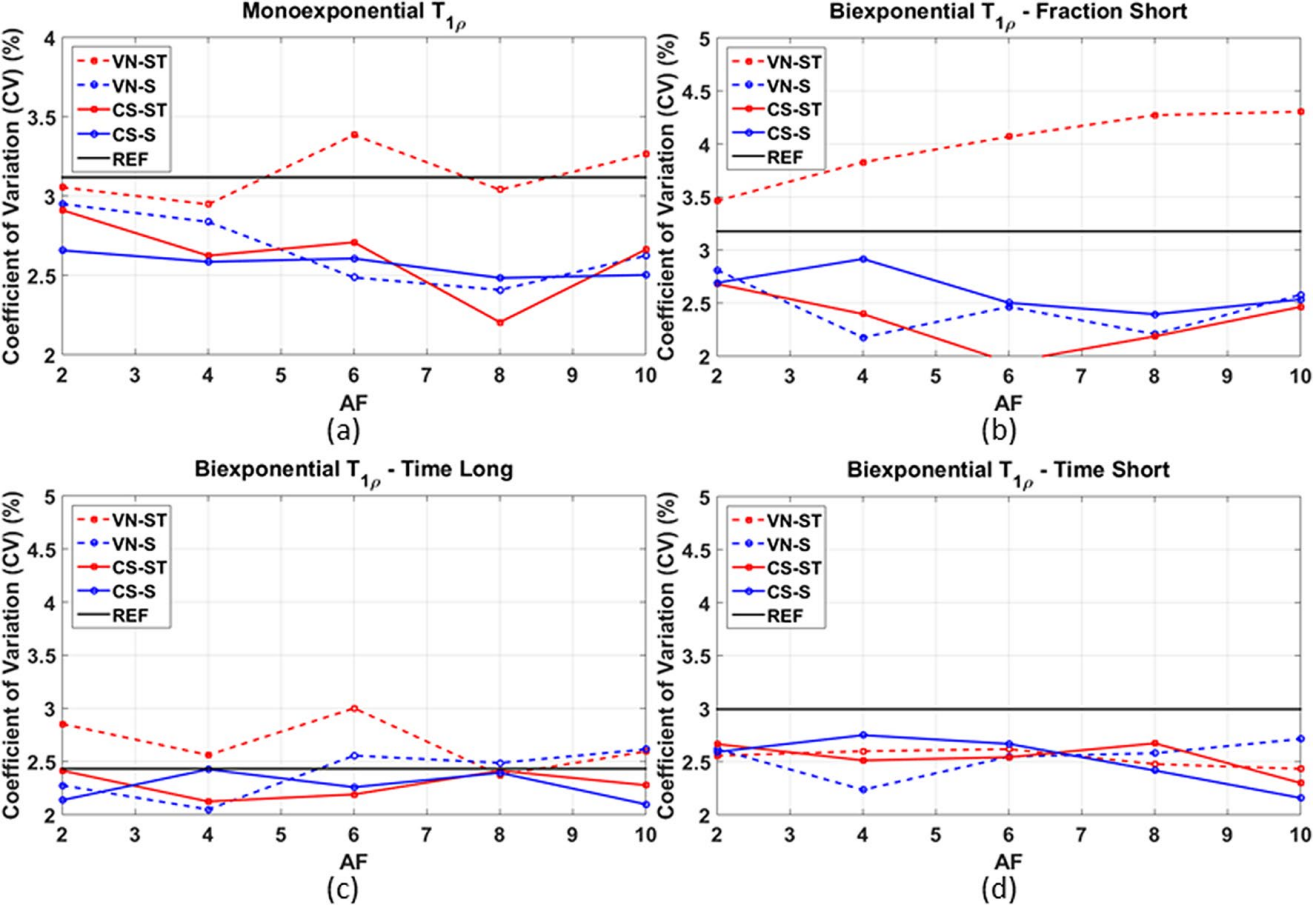
Coefficient of variations of the repeatability study, considering 3 subjects where the acquisition was repeated on the same day, for (**a**) monoexponential T_1ρ_, (**b**) biexponential short fraction, (**c**) biexponential long time, (**d**) biexponential short time.

The coefficient of variations of different methods per AF, showing the variability of the mean parameters between two scans of the same volunteer, is shown in Fig. 7. Essentially, this in vivo analysis of knee cartilage shows that the differences observed in the parameters obtained by each method on the same volunteer when the scan is repeated are between 1.5 and 4.5%. The VN-ST had a larger difference for the fraction of the short component, a little larger than the fully sampled reference (REF). Nevertheless, all methods have shown good repeatability with less than 4.5% difference in the parameters between repeated scans with the same volunteer.

## Discussion

In general, the use of VN is advantageous over CS, even though both approaches provide very satisfying quality for most AF. However, VN provided better reconstruction quality, faster image reconstruction speed, and better biexponential mapping quality. The only drawback is the slightly worse repeatability.

One of the novelties in this study is the use of spatio-temporal information within the VN. The VN-ST improved reconstruction and fitting comparing with the use of only spatial information by VN-S. The VN-ST improved the nRMSE over the VN-S by nearly 9% and improved over CS-ST by nearly 12%.

The training of the regularization parameter in CS, via the coefficient β in ${\varvec{\lambda}}= \beta {\Vert {{{\varvec{C}}}^{\boldsymbol{*}}{{\varvec{F}}}^{\boldsymbol{*}}{\varvec{S}}}^{\boldsymbol{*}}{\varvec{y}}\Vert }_{\infty }$, provided a good automatic adjustment of the regularization parameter. This perhaps helps to solve one of the long-standing questions related to regularizing side penalties, i.e. how to choose the regularization parameter. The training process was successful for the kind of regularizing penalty we used here.

Our VN methods are implemented in PyTorch and run on a GPU cluster. This is necessary to reduce the computational time of the training process. The CS algorithms are implemented in MATLAB and run on a CPU cluster. The average times for the parallel reconstruction of one data set (256 slices) are 110.5 s for CS-S and 133.6 s for CS-ST (CPU cluster composed by Intel i7-1.6 GHz-48 GB machines) and 8.0 s for both, VN-S and VN-ST (GPU cluster composed by NVIDIA M40-12 GB). This means a speedup of 13 times in the image reconstruction time for the VN compared to CS. Note that CS took on average 150 iterations to converge (max iterations was set to 600), while VN is equivalent to 10 iterations (or 10 layers), this means that both methods take roughly 0.8 s/iteration or 0.8 s/layer of processing time. It is expected a computation cost per iteration of the same order for both methods, which indicates that the computation advantage of VN is the small number of iterations (or layers) to achieve a good solution.

CS has been used in monoexponential T_1ρ_ mapping before, some examples are combined CS and auto-calibration reconstruction (ARC)^9^; integrated PCA and dictionary learning (PANDA)^23^ (which was compared to focal underdetermined system solver with PCA (k–t FOCUSS-PCA)^24^ and model-based dictionary learning (MBDL)^25^ in^23^); combined reconstruction with locally adaptive iterative support detection (k–t LAISD) and joint image reconstruction and sensitivity estimation in SENSE (JSENSE)^10^; and blind compressed sensing (BCS)^26^.

Convolutional neural networks^27^ have been used to directly estimate monoexponential T_2_ mapping parameters, with promising results of 6.1% of error in AF of 5 and 7.1% in AF of 8. In^22^ accelerated biexponential T_1ρ_ mapping for brain images has improved L + S methods, but also confirming the need for improvement of biexponential mapping due to model instability.

In^11,12^ we provide a broad evaluation, using AF from 2, up to 10, comparing twelve CS methods, with and without pre-filtering, for mono and biexponential T_1ρ_ mapping. For monoexponential fitting, we cannot see much of an improvement compared with^11^. Considering the results without filtering from^11^, we notice nearly the same level of MNAD per AF. The results in^11^ (Figure 6B, page 1483) are a little better for higher AF due to the optimized choice of the regularization parameter, independent for each image sequence. Here, in Table 1A, we observed an MNAD around 5.0% for AF of 2, which increases almost linearly with the AF to an MNAD of 13.0% for AF of 8, with very little difference between VN-ST, VN-S, and CS-ST.

However, the results here for biexponential mapping are much better than the ones in^12^. One of the factors is the use of complex-valued fitting. In^12^, where magnitude-only fitting was used, the CS-ST (specified as STFD in^12^) has MNAD of 11.4% at AF = 2, and 19.0% at AF = 6. Here, complex-valued fitting was used, according to Table 1B the CS-ST has MNAD of 7.7% at AF = 2 and 13.2% at AF = 6, improving MNAD by around 40% on average when comparing to what was observed in^12^ (Table 2A, page 876). In this sense, VN-ST improved the results obtained by the best CS method even more. The VN-ST has MNAD of 7.4% at AF = 2 and 12.0% at AF = 6, improving MNAD over CS-ST by nearly 7.5% on average. In our recent study in^21^ it was shown that changing the kind of acquisition from Cartesian to golden angle radial also improves CS-ST (STFD in Tables 3A of^21^). This indicates that combining VN with radial acquisition can be a promising future approach.

Based on the results of Table 1, the VN-ST was able to reduce the error on biexponential mapping due to scan acceleration. Using VN-ST, biexponential mapping can be done with an error of 12% at AF = 6, very close to the error of nearly 11% at AF = 6 on monoexponential mapping (achieved by VN-ST, VN-S, and CS-ST). This is an important achievement for biexponential mapping using Cartesian acquisitions that previously had much higher errors compared to monoexponential mapping^11,12,21^.

In the meta-analysis in^1^, considering a pooling of several studies with biomarkers for osteoarthritis (OA), it was observed that the standardized mean difference of monoexponential T_1ρ_ mapping between OA patients and controls ranged from 0.40 and 1.06. In the Supplementary Tables S1–S5, It is shown that up to AF = 6, none of the methods generated a standardized mean difference larger than 0.06, well below the difference between patients and controls.

This is the first study that investigates the use of the VN approach focused on quantitative parametric mapping. Prior studies of VN^14,16–18^ focused on qualitative imaging. This is an important aspect to investigate, because deep learning methods for image reconstruction, in general, could hallucinate structures or alter the temporal behavior of the signals^28,29^, affecting the produced quantitative maps. In this sense, the VN performed very well, producing quantitative parameters consistent with the ones produced by fully-sampled methods.

The number and distribution of TSLs are important. The choice of this work was based on a previous study^3^, but different distributions can be used. Also, the use of different AFs at each time point (or TSL) will be investigated in future studies.

This study evaluates the performance of VN, which is an image reconstruction algorithm based on deep learning. However, the following exponential fitting step is still an optimization step, based on the CGSTR algorithm, not a deep learning-based fitting. In the future, we plan to investigate neural networks also for the fitting task.

The spatio-temporal filters of the VN-ST tested in this study was only of size 11 $\times$ 11 $\times$ 3 due to memory limitation (NVIDIA M40-12 GB). Larger 3D convolutional filters would require GPUs with more memory than what we have available at the moment, but it could significantly improve quality in future implementations of VN-ST.

## Methods

**Figure 8:**
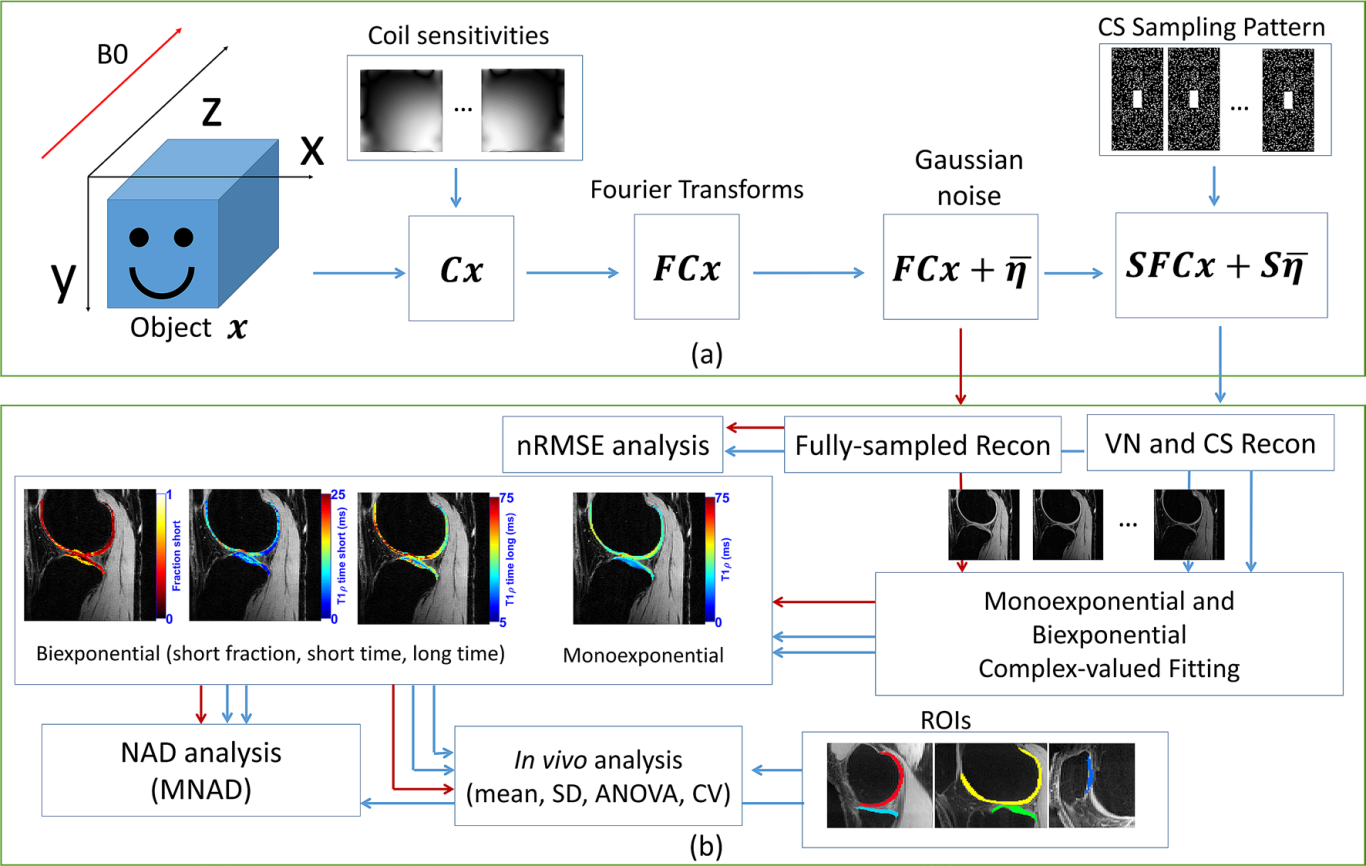
(**a**) MRI acquisition model used for VN and CS reconstructions, including coil sensitivities, Fourier transforms, k-space sampling pattern (using Poisson disk with a fully-sampled central area), and additive white Gaussian noise. (**b**) Diagram of the process, including reconstruction, complex-valued fitting, reconstruction (nRMSE), and fitting (MNAD) error analysis and in vivo data analysis.

Here we detail the methods used in this work, with an overview of them shown in Fig. 8.

### Data acquisition

This study was approved by New York University Langone Health’s institutional review board (IRB) and was health insurance portability and accountability act (HIPAA) compliant. This was a retrospective, non-randomized imaging study to obtain accelerated proton T1ρ relaxation mapping. All subjects gave written informed consent after explanation of the study and the protocol, as per the IRB guidelines. All the methods reported in this manuscript were performed in accordance with the institutional guidelines and regulations.

Seven in vivo human knee 3D-T_1ρ_-weighted datasets were acquired with 10 different spin-lock times (TSLs) using a modified 3D Cartesian Turbo-Flash sequence^3^. The MRI scans were performed using a 3 T clinical MRI scanner (Prisma, Siemens Healthcare, Erlangen, Germany) with a 15-channel Tx/Rx knee coil (QED, Cleveland OH). The 3D-T_1ρ_ acquisition parameters were: TR/TE = 7.5 ms/4 ms, flip angle = 8°, 3D matrix size 256 × 128 × 64 (${N}_{x}\times {N}_{y}\times {N}_{z}$), longitudinal magnetization restoration delay = 1020 ms, 64 k-space lines (${k}_{z}$) captured per preparation pulse, spin-lock frequency = 500 Hz, slice thickness = 2 mm, field of view (FOV) = 120 mm × 120 mm, and receiver bandwidth = 515 Hz/pixel. The readout direction (${k}_{x}$) is always fully sampled (256 samples). The 3D matrix is separated using FFT into multiple 2D problems, and then reorganized with the 10 TSLs into 256 problems using 2D + times systems of size 128 × 64 × 10 (${N}_{y}\times {N}_{z}\times {N}_{t}$).

The T_1ρ_-weighted scans of the knee were acquired in the sagittal plane from seven healthy volunteers (age = 29.6 ± 7.5 years), with 10 TSLs 2/4/6/8/10/15/25/35/45/55 ms, and a total acquisition time of 32 min. The T_1ρ_-protocol was repeated on three volunteers for repeatability evaluation.

### Synthetically generated data

We also generated six synthetic knee 3D-T_1ρ_-weighted datasets, where the T_1ρ_ decaying and intensities are known exactly and used as ground truth. The synthetic data were created using previously obtained T_1ρ_ maps from in vivo knee images. After estimating relaxation maps for all voxels, which were assumed as ground truth, new images and k-space data were synthetically produced, using previously estimated coil sensitivities. In this sense, they are real knee 3D-T_1ρ_-weighted images and not geometric-shaped phantoms. This was done such that the spatial description of the synthetic images is similar to real knee images. However, three synthetic sequences have Gaussian noise added in the k-space, similar to the level of noise observed in our in vivo data acquisition (within the noise calibration acquisitions), while the other three sequences are noiseless. These data will be used to evaluate the performance of the methods in these two scenarios.

### Retrospective undersampling

The 2D + time k-space data were retrospectively undersampled along the two-phase encoding dimensions (k_y_ and k_z_). As mentioned before, the readout, or frequency encoding, direction k_x_ is always fully-sampled in this protocol and it was separated after applying 1D Fourier transform. Data were undersampled following a 2D + time Poisson disk random pattern^30^. The acceleration factor (AF) is defined as the ratio of total k-space samples by the number of measured k-space samples. A central rectangular k-space area (39 × 19 for all AF) was fully sampled and used for coil sensitivity map estimation and low-order phase estimation^31,32^.

### Fully-sampled reference reconstruction

Assuming the k-space data is generated by the model given by

1 $${\varvec{y}}={\varvec{F}}{\varvec{C}}{\varvec{x}}+{\varvec{\eta}},$$

where ${\varvec{x}}\in {\mathbb{C}}^{{N}_{y}{N}_{z}{N}_{t}}$ is a vector that represents the reconstructed image sequence, originally of size ${N}_{y}\times {N}_{z}\times {N}_{t}$, with ${N}_{y}$ being the image size in the y-axis and ${N}_{z}$ the size in the z-axis, ${N}_{t}$ is the number of TSLs. ***y*** is a vector that represents the captured k-space. For these Cartesian acquisitions, the original size of ${\varvec{y}}\in {\mathbb{C}}^{{N}_{y}{N}_{z}{N}_{t}{N}_{c}}$ is ${N}_{y}\times {N}_{z}\times$ ${N}_{t}\times {N}_{c}$, where ${N}_{c}$ is the number of receive coils, and ${\varvec{\eta}}$ represents the Gaussian white noise. The matrix ${\varvec{C}}$ contains the coil sensitivities and phase compensation^31,32^. This matrix maps the image sequence of size ${N}_{y}\times {N}_{z}\times {N}_{t}$ into multiple sensitivity-weighted image sequences of size ${N}_{y}\times {N}_{z}\times {N}_{t}\times {N}_{c}$, followed by a Fourier transform ${\varvec{F}}$.

The reference reconstruction $\widehat{{\varvec{x}}}$ was obtained by applying the adjoint operator:

2 $$\widehat{{\varvec{x}}}={{\varvec{C}}}^{\boldsymbol{*}}{{\varvec{F}}}^{\boldsymbol{*}}{\varvec{y}},$$

where the matrix ${{\varvec{C}}}^{\boldsymbol{*}}$, the adjoint of ${\varvec{C}}$, performs the coil combination and phase compensation, and ${{\varvec{F}}}^{\boldsymbol{*}}$ is the inverse Fourier transform. The coil sensitivities, required for reconstruction, were estimated with ESPIRiT^33^, using the central area of the k-space (39 × 19 for all AF). We also used low-order phase information, following^31,32^, for phase compensation. Phase compensation assures the reconstructed images have nearly zero-phase for later complex-valued fitting in the T_1ρ_ mapping step.

### VN reconstruction algorithms

The VN is inspired by the minimization problem^34^, given by

3 $$\widehat{{\varvec{x}}}\in \underset{{\varvec{x}}}{\mathrm{arg min}}{\Vert {{\varvec{y}}}_{{\varvec{S}}}-{\varvec{S}}{\varvec{F}}{\varvec{C}}{\varvec{x}}\Vert }_{2}^{2}+{\sum }_{i=1}^{{N}_{k}}\langle {\Phi }_{i}\left({{\varvec{K}}}_{{\varvec{i}}}{\varvec{x}}\right),1\rangle ,$$

but instead, it approximates a solution by M fixed iterations of a gradient descent-like algorithm^14^, given by

4 $${{\varvec{x}}}_{m+1}={{\varvec{x}}}_{m}-\left({\sum }_{i=1}^{{N}_{k}} {{\varvec{K}}}_{m,i}^{*}{\Phi }_{m,i}^{^{\prime}}\left({{\varvec{K}}}_{m,i}{{\varvec{x}}}_{m}\right)+{{\alpha }_{m}{{\varvec{C}}}^{\boldsymbol{*}}{{\varvec{F}}}^{\boldsymbol{*}}{\varvec{S}}}^{\boldsymbol{*}}\left({{\varvec{y}}}_{{\varvec{S}}}-{\varvec{S}}{\varvec{F}}{\varvec{C}}{{\varvec{x}}}_{m}\right)\right),$$

where the vector ***x***, matrices ***C*** and ***F*** are described in Eq. (1). The undersampling matrix ***S*** is a diagonal matrix, where the non-sampled k-space points have zeros in their diagonal positions; the respective elements in ${{\varvec{y}}}_{{\varvec{S}}}$ are replaced by zeros as well, being ${{\varvec{y}}}_{{\varvec{S}}}={\varvec{S}}{\varvec{y}}$. Also, $1\le m\le M+1$ represents the iteration index (or layer), where a maximum of $M=10$ was chosen.

All the VN parameters, i.e. convolutional filters ${{\varvec{K}}}_{m,i}$ (${N}_{k}=24$), activation functions ${\Phi }_{m,i}^{^{\prime}}$, and step-sizes ${\alpha }_{m}$, are learned from data^14^. The VN with spatial filters only (VN-S) uses filters of size 11 $\times$ 11, and the VN with spatio-temporal filters (VN-ST) uses filters of size 11 $\times$ 11 $\times$ 3.

Note that the VN in Eq. (4) resembles a general regularized reconstruction algorithm. The left term in (4), ${{\alpha }_{m}{{\varvec{C}}}^{\boldsymbol{*}}{{\varvec{F}}}^{\boldsymbol{*}}{\varvec{S}}}^{\boldsymbol{*}}({{\varvec{y}}}_{{\varvec{S}}}-{\varvec{S}}{\varvec{F}}{\varvec{C}}{{\varvec{x}}}_{m})$, is responsible for reducing k-space error, while the right term in (4), ${\sum }_{i=1}^{{N}_{k}} {{\varvec{K}}}_{m,i}^{*}{\Phi }_{m,i}^{^{\prime}}\left({{\varvec{K}}}_{m,i}{{\varvec{x}}}_{m}\right)$, reduces undesired features in the image. However, instead of using human-designed spatio-temporal filters and activation functions (such components are obtained from the gradient of the cost function in CS methods), the VN uses convolutional filters ${{\varvec{K}}}_{m,i}$ and functions ${\Phi }_{m,i}^{^{\prime}}$ learned from training data. These components are different for each layer $m$.

### CS reconstruction algorithms

In this work, we used one of the best performing regularization functions from^11,12^ for CS of T_1ρ_ mapping of knee cartilage, i.e. the *l*_1_-norm with spatio-temporal finite differences (STFD). This CS reconstructed method is denoted as CS-ST in this paper. We also included spatial finite difference (SFD), to compare CS with the spatial-only version VN. This spatial-only CS reconstructed method is denoted as CS-S.

The *l*_1_-norm^31^ regularized CS problems are posed as

5 $$\widehat{{\varvec{x}}}\in \underset{{\varvec{x}}}{\mathrm{arg min}}{\Vert {{\varvec{y}}}_{{\varvec{S}}}-{\varvec{S}}{\varvec{F}}{\varvec{C}}{\varvec{x}}\Vert }_{2}^{2}+{\varvec{\lambda}}{\Vert {\varvec{T}}{\varvec{x}}\Vert }_{1},$$

The *l*_1_-norm, ${\Vert {\varvec{u}}\Vert }_{1}={\sum }_{i}\left|{u}_{i}\right|$, is the sum of the magnitudes, ${\varvec{\lambda}}$ is the regularization parameter and *i* denotes the pixel index. The transform ***T*** is the SFD, of order 1, or the STFD^35–37^ set to order 1 spatially and order 2 temporally. The CS reconstruction was performed using the new monotone fast iterative shrinkage-thresholding algorithms with variable acceleration (MFISTA-VA)^38^. All methods stopped when ${\Vert {{\varvec{x}}}_{i+1}-{{\varvec{x}}}_{i}\Vert }_{2}/{\Vert {{\varvec{x}}}_{i+1}\Vert }_{2}<{10}^{-5}$, or when $i>600$, $i$ the iteration index.

### Training VN parameters and regularization parameter of CS

The datasets, composed of real data and synthetically generated data were divided into two groups. One group is used for training ($n=4$ real datasets, $n=3$ synthetically generated datasets, each dataset contains 256 slices of 2D + time T_1ρ_ image sequences), and the other group is used for testing ($n=3$ real datasets, $n=3$ synthetically generated datasets). The training set totals $(4+3)\times 256=3072$ image sequences for training the algorithms.

The training of the VN parameters was done using 50 epochs of the algorithm ADAM^39^, where the error $\sum_{j=1}^{J}{\Vert {{\varvec{x}}}_{M,j}-{{\varvec{x}}}_{ref,j}\Vert }_{2}^{2}$ is minimized. The error with validation set (subset of the training data randomly chosen) stopped to decreasing at this iteration. The batch size for VN-S is 40 image sequences, and for VN-ST is 20 image sequences. These batch sizes are the largest that can fit into the GPU memory. The learning rate was set to ${10}^{-3}$, the recommended for ADAM^39^. The vector ${{\varvec{x}}}_{M,j}$ is the VN reconstruction of the *j*th image sequence in the training set, and ${{\varvec{x}}}_{ref,j}$ is either the ground truth (if the *j*th image is from the synthetic dataset) or the fully-sampled reconstruction, from Eq. (2) (if the *j*th image is from the real dataset) for the same image sequence. The step parameters ${\alpha }_{m}$, convolutional filters ${{\varvec{K}}}_{m,i}$, and activation functions ${\Phi }_{m,i}^{^{\prime}}$ are learned during the training process.

For CS reconstructions, the training set was used to find the regularization parameters, ${\varvec{\lambda}}$, from Eq. (5). The parameters related to the *l*_1_-norm for each dataset was set to ${\varvec{\lambda}}= \beta {\Vert {{{\varvec{C}}}^{\boldsymbol{*}}{{\varvec{F}}}^{\boldsymbol{*}}{\varvec{S}}}^{\boldsymbol{*}}{\varvec{y}}\Vert }_{\infty }$, where $\beta$ is chosen such the error $\sum_{j=1}^{J}{\Vert {\widehat{{\varvec{x}}}}_{\beta ,j}-{{\varvec{x}}}_{ref,j}\Vert }_{2}^{2}$ is minimized where ${\widehat{{\varvec{x}}}}_{\beta ,j}$ is the CS reconstruction of the *j*th image sequence in the set. The best parameter $\beta$ searched among 12 log spaced coefficients between ${10}^{-6}$ and ${10}^{6}$ (multiplicative factor of 12.3285), with an extra 12 steps for refinement using bisection among the best coefficients.

### Exponential models and fitting algorithms

The T_1ρ_ relaxation is assumed to be an exponentially decaying process. However, the measured magnitude of the signals only shows this decaying behavior in noise-free cases. When the signal is contaminated by noise, such as Gaussian noise, the magnitude decaying converges to a non-zero constant (a bias) due to Rician statistics^40,41^. The difficulty associated with this approach of using the magnitude-only fitting, such as the ones used in^11,12^, is that the kind of noise and the levels of noise in the images are different according to the kind of reconstruction method utilized (i.e. VN or CS), requiring individualized compensation. Here, we decided to use a different approach, using complex-valued fitting^41,42^. In this case, noise is not expected to cause bias^41^, increasing the accuracy of the estimated exponential parameters over real-valued fitting using only the magnitude.

The complex-valued monoexponential model is described as

6 $$x(t,{\varvec{n}})=c({\varvec{n}})\mathit{exp}\left(-\frac{t}{\tau ({\varvec{n}})}\right),$$

with complex-valued $c({\varvec{n}})$. Note the relaxation time $\tau ({\varvec{n}})$ is real-valued.

The complex-valued biexponential model can be written as:

7 $$x\left(t,{\varvec{n}}\right)=c\left({\varvec{n}}\right)\left({f}_{s}\left({\varvec{n}}\right)\mathit{exp}\left(-\frac{t}{{\tau }_{s}\left({\varvec{n}}\right)}\right)+{f}_{l}\left({\varvec{n}}\right)\mathit{exp}\left(-\frac{t}{{\tau }_{l}\left({\varvec{n}}\right)}\right)\right),$$

where $c({\varvec{n}})$ is complex-valued. However, the fractions of short and long components at position ***n***, given by ${0\le f}_{s}({\varvec{n}})\le 1$ and ${f}_{l}\left({\varvec{n}}\right)=1-{f}_{s}({\varvec{n}})$, and the T_1ρ_ relaxation times of the short and long components, given by ${\tau }_{s}({\varvec{n}})$ and ${\tau }_{l}({\varvec{n}})$, are all real-valued.

The biexponential T_1ρ_ parameters estimation, or simply fitting process, was done using non-linear least squares, using models of Eqs. (6) and (7), where the minimization was done using conjugate gradient Steihaug’s trust-region (CGSTR) algorithm^43^. The CGSTR algorithm stopped at a maximum of 2000 iterations for monoexponential, or 4000 iterations for biexponential, or else when normalized parameter update is lower than 10^–5^. Biexponential estimation started with monoexponential fitting results, classifying them as short (0.5-10 ms) or long (10-300 ms), depending on its estimated monoexponential T_1ρ_ relaxation time. Similar to^44^, F-test was utilized for detecting mono/biexponential voxels. Voxels were assumed to have biexponential behavior if F-ratio > 5.14 (*p* value = 0.05) related to monoexponential, following the F-test method from^45^. This means that the sum of squares of the biexponential fitting process is reduced significantly compared to monoexponential fitting. Also, both fractions ${f}_{s}({\varvec{n}})$ and ${f}_{l}\left({\varvec{n}}\right)$ need to be higher than 5% in order to be a valid biexponential in these experiments. voxels that did not satisfy F-ratio > 5.14 or minimum fraction of 5% were excluded from biexponential evaluations.

### Analysis of the reconstructions

The performance of the VN and CS methods was evaluated according to the quality of the reconstructed images and the quality of the estimated T_1ρ_ parameters. Image reconstruction quality was assessed using normalized root mean squared error (nRMSE) against the reference (REF) method. Our reference method is the reconstruction of the fully-sampled data or the ground truth (for the synthetic phantom). When the ground truth is available, the fully-sampled reconstruction was also compared and plotted as REF, however, no acceleration is applied to it. The nRMSE is defined as

8 $$nRMSE\left(\widehat{{\varvec{x}}},{{\varvec{x}}}_{\mathrm{ref}}\right)=\frac{{\Vert \widehat{{\varvec{x}}}-{{\varvec{x}}}_{\mathrm{ref}}\Vert }_{2}}{{\Vert {{\varvec{x}}}_{\mathrm{ref}}\Vert }_{2}}.$$

### Analysis of the fitting

**Figure 9:**
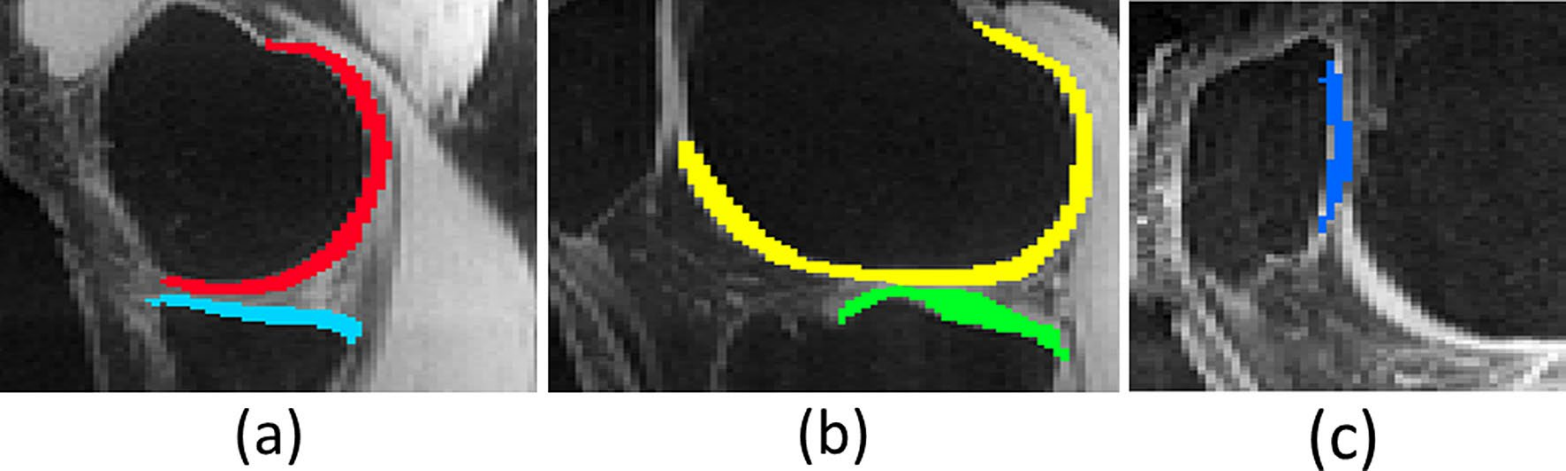
The regions of interest (ROIs) consider: (**a**) medial femoral cartilage (MFC), in red, and medial tibial cartilage (MTC), in cyan; (**b**) lateral femoral cartilage (LFC), in yellow, and lateral tibial cartilage (LTC), in green; and (**c**) patellar cartilage (PC), in blue. In this figure only one slice is shown, each ROI extends across five slices.

The fitting process was applied only on each specific ROI, as shown in Fig. 9. For in vivo knee cartilage, 5 ROIs were employed, following^3^: medial femoral cartilage (MFC), medial tibial cartilage (MTC), lateral femoral cartilage (LFC), lateral tibial cartilage (LTC), and patellar cartilage (PC). In those regions, the T_1ρ_ parameters from CS and VN reconstructions, including T_1ρ_ times and fractions for short and long components, were compared against the parameters obtained from the reference reconstruction (and ground truth, when available).

The quality was assessed using normalized absolute deviation (NAD) of the parameters^11,12^ obtained in each voxel position ***n***, given by

9 $$NAD\left({\varvec{n}}\right)=\frac{\left|p\left({\varvec{n}}\right)-{p}_{ref}\left({\varvec{n}}\right)\right|}{(p\left({\varvec{n}}\right)+{p}_{ref}\left({\varvec{n}}\right))/2},$$

where *p*(***n***) is the T_1ρ_ time for the monoexponential model in Eq. (6) or one of the four biexponential parameters (${f}_{s}\left({\varvec{n}}\right),{f}_{l}\left({\varvec{n}}\right),{\tau }_{s}\left({\varvec{n}}\right), {\tau }_{l}({\varvec{n}})$) for Eq. (7). Voxels in which any of the fractions were lower than 5% were excluded from the biexponential evaluation. As observed here and in^3^, small fractions had inaccurate estimated T_1ρ_ parameters, even for fully-sampled images, leading to unrealistic NADs.

The errors in T_1ρ_ mapping were quantified by the median of NADs (MNAD):

10 $$MNAD({\varvec{R}}{\varvec{O}}{\varvec{I}})=\underset{{\varvec{n}}\in {\varvec{ROI}}}{\mathrm{median}}\left(\frac{\left|p\left({\varvec{n}}\right)-{p}_{ref}\left({\varvec{n}}\right)\right|}{(p\left({\varvec{n}}\right)+{p}_{ref}\left({\varvec{n}}\right))/2}\right),$$

The ROI can comprehend a specific ROI as shown in Fig. 9, or all ROIs. In Eq. (10), MNAD of 0.1 corresponds to a median deviation of 10% on the parameters relative to the average between reference and evaluated value.

### In vivo statistical data analysis

In order to compare *in-vivo* quantitative parameters among different subjects and acquisition, where voxel-based metrics are not possible, we used mean parameters of an ROI, given by

11 $$\stackrel{-}{p}\left({\varvec{R}}{\varvec{O}}{\varvec{I}}\right)=\underset{{\varvec{n}}\in {\varvec{R}}{\varvec{O}}{\varvec{I}}}{\mathrm{mean}} p\left({\varvec{n}}\right).$$

The mean, in Eq. (11), is used as a measurement of central tendency of the parameters of the relaxation model (e.g., times and fractions) in the ROI. The variability of relaxation parameters are measured by the standard deviation (SD), given by

12 $$SD\left({\varvec{R}}{\varvec{O}}{\varvec{I}}\right)=\sqrt{\frac{1}{{N}_{ROI}}\sum_{{\varvec{n}}\in {\varvec{R}}{\varvec{O}}{\varvec{I}}}{\left|p\left({\varvec{n}}\right)-\stackrel{-}{p}\left({\varvec{R}}{\varvec{O}}{\varvec{I}}\right)\right|}^{2}} .$$

Analysis of variance (ANOVA) is used to evaluate if the differences between estimated $\stackrel{-}{p}\left({\varvec{R}}{\varvec{O}}{\varvec{I}}\right)$ , from Eq. (11), for the various accelerated methods are greater than would be expected by chance. This is shown by the *p* value  for a balanced one-way ANOVA in the Supplementary Tables S1–S5. The averaged $\stackrel{-}{p}\left({\varvec{R}}{\varvec{O}}{\varvec{I}}\right)$ and $SD\left({\varvec{R}}{\varvec{O}}{\varvec{I}}\right)$ across different volunteers are shown in Fig. 6. Supplementary Tables S1–S5 also show the standardized mean difference to compare with results in^1^.

Intra-subject repeatability is assessed using the coefficient of variation (CV), defined as CV = SD/M, being SD the standard deviation and M the average of $\stackrel{-}{p}\left({\varvec{R}}{\varvec{O}}{\varvec{I}}\right)$ of two scans of the same volunteer.

## Supplementary information

Supplementary Information

## Data Availability

The datasets generated during and/or analyzed during the current study are available from the corresponding author on reasonable request.
