# Supplementary material for: Rapid mono and biexponential 3D-T1ρ mapping of knee cartilage using variational networks
Source: Sci Rep. 2020 Nov 5;10:19144. doi: 10.1038/s41598-020-76126-x (PMC7645759; doi:10.1038/s41598-020-76126-x)
Supplement: Supplementary file 1 — Supplementary Information [file 41598_2020_76126_MOESM1_ESM.docx]

Supplemental information for the paper:

**Rapid Mono and Biexponential 3D-T1ρ Mapping of Knee Cartilage Using Variational Networks**

**Authors:** Marcelo V W Zibetti^1^, Patricia M Johnson^1^, Azadeh Sharafi^1^, Kerstin Hammernik^2^, Florian Knoll^1^, and Ravinder R Regatte^1^.

**Affiliations:**

*^1^ Bernard and Irene Schwartz* *Center for Biomedical Imaging, Department of Radiology, New York University School of Medicine, New York, NY, USA*

*^2^ Department of Computing, Imperial College London, London, United Kingdom*

*Correspondence to:

Marcelo V W Zibetti, Dr. Eng.

New York University School of Medicine

Bernard and Irene Schwartz Center for Biomedical Imaging

660 1^st^ Ave, 4^th^ Floor

New York, NY-10016, USA

Phone: +1-646-501-9338

Email: Marcelo.WustZibetti@nyulangone.org

This additional supporting information may be found in the online version of this article, including:

- Supporting Information Tables S1 to S5 for Results

**Supporting Information Table S1:** Central tendency (mean, equation (11)) and variability (standard deviation (SD), equation (12)) of monoexponential T_1ρ_ values (in ms) in different ROI of the cartilage (according to regions observed in Figure 2) for the reference method, VN and CS methods with AF=4 and AF=8. Strictly standardized mean difference (SSMD) is also shown.

|  |  |  | **AF=4** | | | | **AF=8** | | | |
| --- | --- | --- | --- | --- | --- | --- | --- | --- | --- | --- |
|  |  | **REF** | **VN-ST** | **VN-S** | **CS-ST** | **CS-S** | **VN-ST** | **VN-S** | **CS-ST** | **CS-S** |
| **MFC** | **mean** | 36.5 | 36.4 | 36.1 | 35.6 | 35.2 | 36.5 | 35.8 | 35.5 | 35.2 |
| **p=0.81** | **SD** | 17.3 | 14.9 | 16.8 | 15.6 | 14.3 | 14.8 | 15.1 | 14.7 | 13.9 |
| **MTC** | **mean** | 31.8 | 32.0 | 31.7 | 31.1 | 30.8 | 32.0 | 31.7 | 31.0 | 30.9 |
| **p=0.88** | **SD** | 14.4 | 13.9 | 14.2 | 12.3 | 11.2 | 11.8 | 13.2 | 11.7 | 11.0 |
| **LFC** | **mean** | 40.1 | 39.5 | 39.4 | 39.2 | 38.6 | 39.5 | 39.1 | 38.8 | 38.4 |
| **p=0.80** | **SD** | 28.8 | 24.6 | 26.7 | 26.8 | 25.4 | 23.2 | 26.2 | 25.7 | 24.6 |
| **LTC** | **mean** | 29.5 | 29.3 | 29.1 | 29.1 | 28.7 | 29.2 | 28.8 | 28.9 | 28.6 |
| **p=0.99** | **SD** | 16.2 | 14.9 | 14.7 | 14.6 | 13.3 | 12.4 | 13.3 | 13.4 | 12.2 |
| **PC** | **mean** | 41.2 | 40.7 | 40.8 | 40.1 | 39.6 | 40.7 | 40.1 | 40.0 | 39.4 |
| **p=0.99** | **SD** | 24.6 | 18.6 | 22.9 | 21.7 | 20.1 | 16.4 | 20.4 | 20.4 | 17.2 |
| **ALL** | **mean** | 37.1 | 36.8 | 36.7 | 36.4 | 35.8 | 36.8 | 36.4 | 36.1 | 35.7 |
| **p=0.84** | **SD** | 24.2 | 20.6 | 22.8 | 22.1 | 20.8 | 19.2 | 21.5 | 21.0 | 19.6 |
|  | **SSMD** |  | **0.01** | **0.01** | **0.02** | **0.04** | **0.01** | **0.02** | **0.03** | **0.04** |

**Supporting Information Table S2:** Central tendency (mean, equation (11)) and variability (standard deviation, equation (12)) of Biexponential T_1ρ_ time of the short component (in ms) in different ROI of the cartilage (according to regions observed in Figure 2) for the reference method, VN and CS methods with AF=4 and AF=8. Strictly standardized mean difference (SSMD) is also shown.

|  |  |  | **AF=4** | | | | **AF=8** | | | |
| --- | --- | --- | --- | --- | --- | --- | --- | --- | --- | --- |
|  |  | **REF** | **VN-ST** | **VN-S** | **CS-ST** | **CS-S** | **VN-ST** | **VN-S** | **CS-ST** | **CS-S** |
| **MFC** | **mean** | 6.0 | 6.0 | 6.0 | 6.1 | 5.9 | 6.0 | 5.9 | 6.1 | 6.0 |
| **p=0.69** | **SD** | 1.7 | 1.4 | 1.7 | 1.6 | 1.8 | 1.3 | 1.6 | 1.7 | 1.8 |
| **MTC** | **mean** | 5.9 | 5.9 | 5.9 | 6.1 | 5.9 | 5.9 | 5.9 | 6.1 | 6.0 |
| **p=0.99** | **SD** | 1.7 | 1.5 | 1.7 | 1.7 | 1.8 | 1.4 | 1.7 | 1.7 | 1.9 |
| **LFC** | **mean** | 5.8 | 5.8 | 5.8 | 5.9 | 5.8 | 5.8 | 5.8 | 5.9 | 5.8 |
| **p=0.41** | **SD** | 1.6 | 1.4 | 1.6 | 1.6 | 1.8 | 1.3 | 1.6 | 1.6 | 1.8 |
| **LTC** | **mean** | 5.7 | 5.7 | 5.8 | 5.9 | 5.8 | 5.7 | 5.8 | 5.9 | 5.8 |
| **p=0.97** | **SD** | 1.6 | 1.5 | 1.7 | 1.7 | 1.8 | 1.4 | 1.7 | 1.7 | 1.8 |
| **PC** | **mean** | 5.9 | 6.0 | 5.9 | 6.0 | 5.9 | 5.9 | 5.9 | 6.0 | 5.9 |
| **p=0.93** | **SD** | 1.6 | 1.3 | 1.6 | 1.6 | 1.8 | 1.3 | 1.7 | 1.7 | 1.8 |
| **ALL** | **mean** | 5.9 | 5.9 | 5.9 | 6.0 | 5.9 | 5.9 | 5.9 | 6.0 | 5.9 |
| **p=0.88** | **SD** | 1.7 | 1.4 | 1.7 | 1.7 | 1.8 | 1.4 | 1.7 | 1.7 | 1.9 |
|  | **SSMD** |  | **0.01** | **0.01** | **0.05** | **0.00** | **0.01** | **0.01** | **0.05** | **0.00** |

**Supporting Information Table S3:** Central tendency (mean, equation (11)) and variability (standard deviation, equation (12)) of Biexponential T_1ρ_ time of the long component (in ms) in different ROI of the cartilage (according to regions observed in Figure 2) for the reference method, VN and CS methods with AF=4 and AF=8. Strictly standardized mean difference (SSMD) is also shown.

|  |  |  | **AF=4** | | | | **AF=8** | | | |
| --- | --- | --- | --- | --- | --- | --- | --- | --- | --- | --- |
|  |  | **REF** | **VN-ST** | **VN-S** | **CS-ST** | **CS-S** | **VN-ST** | **VN-S** | **CS-ST** | **CS-S** |
| **MFC** | **mean** | 44.0 | 43.6 | 43.4 | 42.4 | 41.9 | 43.8 | 43.0 | 42.2 | 41.8 |
| **p=0.54** | **SD** | 19.9 | 16.3 | 18.9 | 16.7 | 16.1 | 15.9 | 17.6 | 16.9 | 15.5 |
| **MTC** | **mean** | 39.7 | 39.6 | 39.8 | 39.3 | 38.9 | 40.0 | 39.6 | 39.1 | 39.0 |
| **p=0.99** | **SD** | 14.8 | 13.3 | 14.5 | 15.5 | 14.4 | 12.6 | 14.2 | 12.7 | 11.9 |
| **LFC** | **mean** | 49.0 | 47.7 | 48.2 | 47.7 | 46.7 | 48.0 | 48.5 | 47.0 | 46.2 |
| **p=0.33** | **SD** | 32.5 | 27.2 | 31.4 | 30.7 | 29.7 | 26.4 | 32.1 | 30.5 | 28.9 |
| **LTC** | **mean** | 36.8 | 36.4 | 36.5 | 36.2 | 35.8 | 36.6 | 36.6 | 36.2 | 35.6 |
| **p=0.99** | **SD** | 19.3 | 15.4 | 16.8 | 16.7 | 15.7 | 14.8 | 17.0 | 16.4 | 14.8 |
| **PC** | **mean** | 50.2 | 49.0 | 49.9 | 48.5 | 47.6 | 50.0 | 49.0 | 48.4 | 47.4 |
| **p=0.94** | **SD** | 29.1 | 20.6 | 27.3 | 24.7 | 23.1 | 19.8 | 25.4 | 25.2 | 22.2 |
| **ALL** | **mean** | 45.5 | 44.7 | 45.1 | 44.3 | 43.6 | 45.0 | 44.9 | 44.0 | 43.3 |
| **p=0.28** | **SD** | 27.9 | 22.6 | 26.8 | 25.4 | 24.3 | 21.8 | 26.2 | 25.1 | 23.2 |
|  | **SSMD** |  | **0.02** | **0.01** | **0.03** | **0.05** | **0.01** | **0.02** | **0.04** | **0.06** |

**Supporting Information Table S4:** Central tendency (mean, equation (11)) and variability (standard deviation, equation (12)) of Biexponential T_1ρ_ fraction of the short component (in %) in different ROI of the cartilage (according to regions observed in Figure 2) for the reference method, VN and CS methods with AF=4 and AF=8. Strictly standardized mean difference (SSMD) is also shown.

|  |  |  | **AF=4** | | | | **AF=8** | | | |
| --- | --- | --- | --- | --- | --- | --- | --- | --- | --- | --- |
|  |  | **REF** | **VN-ST** | **VN-S** | **CS-ST** | **CS-S** | **VN-ST** | **VN-S** | **CS-ST** | **CS-S** |
| **MFC** | **mean** | 20.7 | 19.4 | 20.6 | 20.2 | 20.4 | 19.3 | 20.3 | 19.9 | 20.2 |
| **p=0.70** | **SD** | 16.9 | 12.6 | 16.8 | 16.6 | 16.2 | 11.2 | 16.6 | 16.0 | 15.4 |
| **MTC** | **mean** | 27.5 | 26.6 | 27.4 | 27.5 | 27.6 | 25.8 | 26.2 | 27.3 | 27.2 |
| **p=0.99** | **SD** | 20.5 | 18.3 | 20.6 | 21.3 | 21.1 | 17.6 | 19.9 | 21.5 | 21.2 |
| **LFC** | **mean** | 21.0 | 19.7 | 20.9 | 20.8 | 20.9 | 19.2 | 20.9 | 20.5 | 20.7 |
| **p=0.88** | **SD** | 17.7 | 13.4 | 17.4 | 17.7 | 17.3 | 11.4 | 16.7 | 17.1 | 16.4 |
| **LTC** | **mean** | 25.9 | 25.5 | 25.9 | 25.5 | 25.6 | 25.3 | 26.1 | 25.3 | 25.3 |
| **p=0.99** | **SD** | 20.2 | 17.9 | 20.2 | 20.5 | 20.3 | 17.2 | 20.4 | 20.3 | 19.6 |
| **PC** | **mean** | 20.0 | 18.3 | 19.9 | 19.7 | 19.9 | 18.3 | 19.6 | 19.5 | 20.1 |
| **p=0.97** | **SD** | 15.0 | 10.3 | 15.0 | 15.5 | 15.3 | 9.1 | 13.8 | 15.5 | 15.6 |
| **ALL** | **mean** | 22.2 | 21.0 | 22.1 | 21.9 | 22.0 | 20.7 | 21.9 | 21.6 | 21.9 |
| **p=0.95** | **SD** | 18.9 | 15.4 | 18.8 | 19.0 | 18.6 | 14.1 | 18.4 | 18.6 | 18.1 |
|  | **SSMD** |  | **0.05** | **0.00** | **0.01** | **0.01** | **0.06** | **0.01** | **0.02** | **0.01** |

**Supporting Information Table S5:** Central tendency (mean, equation (11)) and variability (standard deviation, equation (12)) of Biexponential T_1ρ_ fraction of the long component (in %) in different ROI of the cartilage (according to regions observed in Figure 2) for the reference method, VN and CS methods with AF=4 and AF=8. Strictly standardized mean difference (SSMD) is also shown.

|  |  |  | **AF=4** | | | | **AF=8** | | | |
| --- | --- | --- | --- | --- | --- | --- | --- | --- | --- | --- |
|  |  | **REF** | **VN-ST** | **VN-S** | **CS-ST** | **CS-S** | **VN-ST** | **VN-S** | **CS-ST** | **CS-S** |
| **MFC** | **mean** | 79.3 | 80.6 | 79.4 | 79.8 | 79.6 | 80.7 | 79.7 | 80.1 | 79.8 |
| **p=0.70** | **SD** | 16.9 | 12.6 | 16.8 | 16.6 | 16.2 | 11.2 | 16.6 | 16.0 | 15.4 |
| **MTC** | **mean** | 72.5 | 73.4 | 72.6 | 72.5 | 72.4 | 74.2 | 73.8 | 72.7 | 72.8 |
| **p=0.99** | **SD** | 20.5 | 18.3 | 20.6 | 21.3 | 21.1 | 17.6 | 19.9 | 21.5 | 21.2 |
| **LFC** | **mean** | 79.0 | 80.3 | 79.1 | 79.2 | 79.1 | 80.8 | 79.1 | 79.5 | 79.3 |
| **p=0.88** | **SD** | 17.7 | 13.4 | 17.4 | 17.7 | 17.3 | 11.4 | 16.7 | 17.1 | 16.4 |
| **LTC** | **mean** | 74.1 | 74.5 | 74.1 | 74.5 | 74.4 | 74.7 | 73.9 | 74.7 | 74.7 |
| **p=0.99** | **SD** | 20.2 | 17.9 | 20.2 | 20.5 | 20.3 | 17.2 | 20.4 | 20.3 | 19.6 |
| **PC** | **mean** | 80.0 | 81.7 | 80.1 | 80.3 | 80.1 | 81.7 | 80.4 | 80.5 | 79.9 |
| **p=0.87** | **SD** | 15.0 | 10.3 | 15.0 | 15.5 | 15.3 | 9.1 | 13.8 | 15.5 | 15.6 |
| **ALL** | **mean** | 77.8 | 79.0 | 77.9 | 78.1 | 78.0 | 79.3 | 78.1 | 78.4 | 78.1 |
| **p=0.95** | **SD** | 18.9 | 15.4 | 18.8 | 19.0 | 18.6 | 14.1 | 18.4 | 18.6 | 18.1 |
|  | **SSMD** |  | **0.05** | **0.00** | **0.01** | **0.01** | **0.06** | **0.01** | **0.02** | **0.01** |
